# Accumulation of heavy metals and trace elements in fluvial sediments received effluents from traditional and semiconductor industries

**DOI:** 10.1038/srep34250

**Published:** 2016-09-29

**Authors:** Liang-Ching Hsu, Ching-Yi Huang, Yen-Hsun Chuang, Ho-Wen Chen, Ya-Ting Chan, Heng Yi Teah, Tsan-Yao Chen, Chiung-Fen Chang, Yu-Ting Liu, Yu-Min Tzou

**Affiliations:** 1 Scientific Research Division, National Synchrotron Radiation Research Center, 101 Hsin-Ann Road, Hsinchu 300, Taiwan (R.O.C.); 2 Department of Environmental Science and Engineering, Tunghai University, 1727 Sec.4, Taiwan Boulevard, Taichung 407, Taiwan (R.O.C.); 3 Department of Soil and Environmental Sciences, National Chung-Hsing University, 145 Xingda Rd., Taichung 402, Taiwan (R.O.C.); 4 Division of Environmental Studies, Graduate School of Frontier Sciences, The University of Tokyo, 332 Building of Environmental Studies, 5-1-5 Kashiwanoha, Kashiwa City, Chiba 277-8563, Japan; 5 Department of Engineering and System Sciences, National Tsing Hua University, No. 101, Section 2, Kuang-Fu Road, Hsinchu 300, Taiwan (R.O.C.)

## Abstract

Metal accumulation in sediments threatens adjacent ecosystems due to the potential of metal mobilization and the subsequent uptake into food webs. Here, contents of heavy metals (Cd, Cr, Cu, Ni, Pb, and Zn) and trace elements (Ga, In, Mo, and Se) were determined for river waters and bed sediments that received sewage discharged from traditional and semiconductor industries. We used principal component analysis (PCA) to determine the metal distribution in relation to environmental factors such as pH, EC, and organic matter (OM) contents in the river basin. While water PCA categorized discharged metals into three groups that implied potential origins of contamination, sediment PCA only indicated a correlation between metal accumulation and OM contents. Such discrepancy in metal distribution between river water and bed sediment highlighted the significance of physical-chemical properties of sediment, especially OM, in metal retention. Moreover, we used Se XANES as an example to test the species transformation during metal transportation from effluent outlets to bed sediments and found a portion of Se inventory shifted from less soluble elemental Se to the high soluble and toxic selenite and selenate. The consideration of environmental factors is required to develop pollution managements and assess environmental risks for bed sediments.

Heavy metal distribution has been one of the critical concerns in natural environments due to their toxicity and biomagnification attributes. Lots of regulations have been established to avoid heavy metal concentrations in waters, sediments, and soils exceed quality criteria for environmental protection. Anthropogenic activities have discharged significant amounts of heavy metals into rivers. By accumulated in sediments, heavy metals could enter the food chain as bottom sediments in river basin or reservoirs serve as habitats and food sources for benthic fauna. Subsequently, heavy metals may directly or indirectly threaten the aquatic flora and fauna. Thereby, metal contamination in sediments has been an ongoing issue worldwide^1^ as sediment-bound metals may be of great significance for water quality and ecosystem health^2^,^3^.

According to chemical and geological conditions, heavy metals discharged into surface waters were rapidly partitioned onto particulate matter and incorporated in sediments. Based on the matrix composition, sediments generally show a superior capacity for metal retention^4^. The degree of heavy metals and trace elements retained in sediments is controlled by not only the dynamics of physical-chemical equilibration, which is governed by pH, redox conditions, oxidation states of elements, etc., but also sediment attributes such as particle size and contents for organic matter (OM) as well as Fe/Mn (hydr)oxdies^5^. Most importantly, the relative abundance of heavy metals in sediments is mainly dependent on the waste discharged from industrial and other anthropogenic activities^6^,^7^.

Our study site locates at the basin of the Fazih and the Wu River in Taichung, Taiwan (Fig. 1). This basin has received treated wastewater from the Taichung Industrial Park, the Taichung Precision Machinery Innovation Technology Park, the Central Taiwan Science Park, and untreated wastewater from domestic original equipment manufacturers (OEM). More than 19 million tons of wastewater has been discharged annually into the Fazih and the Wu River. Amounts and types of metals discharged may vary according to sources and intensities of industrial activities. For traditional industrial activities such as printed board manufacturing, metal finishing and electroplating, and textile dyes, the discharged heavy metals generally consist of cadmium (Cd), chromium (Cr), copper (Cu), nickel (Ni), lead (Pb), zinc (Zn)^6^,^8^,^9^,^10^,^11^,^12^, etc. For the semiconductor industries, however, the intensive development of manufacturing processes has embraced a boom in the application of trace elements such as selenium (Se), gallium (Ga), indium (In), and molybdenum (Mo) to improve the performance of thin film growth, chemical polishing, and photolithography.

Here, we aimed to determine how environmental factors such as pH, electrical conductivity (EC), oxidation-reduction potential (ORP), OM, amorphous (oxalate-extractable) aluminum (Al) and (Fe) dominate the accumulation of heavy metals and trace elements in bed sediments. Data sets on environmental factors and metal contents in river water and bed sediments were subjected to principal component analysis (PCA) to ascertain factors that were responsible for the variation in metal loading in sediments. In particular, Se speciation was performed as an example to examine the species transformation in relation to environmental changes by X-ray absorption near edge structure (XANES) analysis during the transportation from effluent outlet to bed sediments. Recognition of the metal enrichment factors could lead to a better estimation of the dynamics and mass balance of heavy metals and trace elements in contaminated sediments and thereby an improved strategy for wastewater discharge management.

## Results

### Environmental parameters

**Figure 1:**
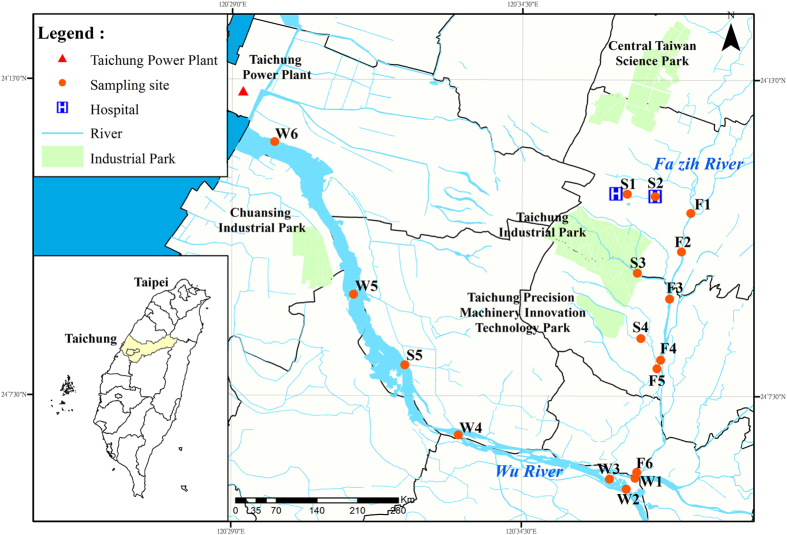
Sampling sites for river waters, sediments, or discharged sewage effluents in the Fazih and the Wu River basin. Water and sediment samples were collected from 12 sampling sites scattered in the Fazih River (F1–F6) and the Wu River (W1–W6). The sewage effluents were discharged from hospitals (S1 and S2), the Taichung Industrial Park (S3), the Taichung Precision Machinery Innovation Technology Park (S4), and the Central Taiwan Science Park (S5). The map was generated using ArcGIS 10.0 software.

**Figure 2:**
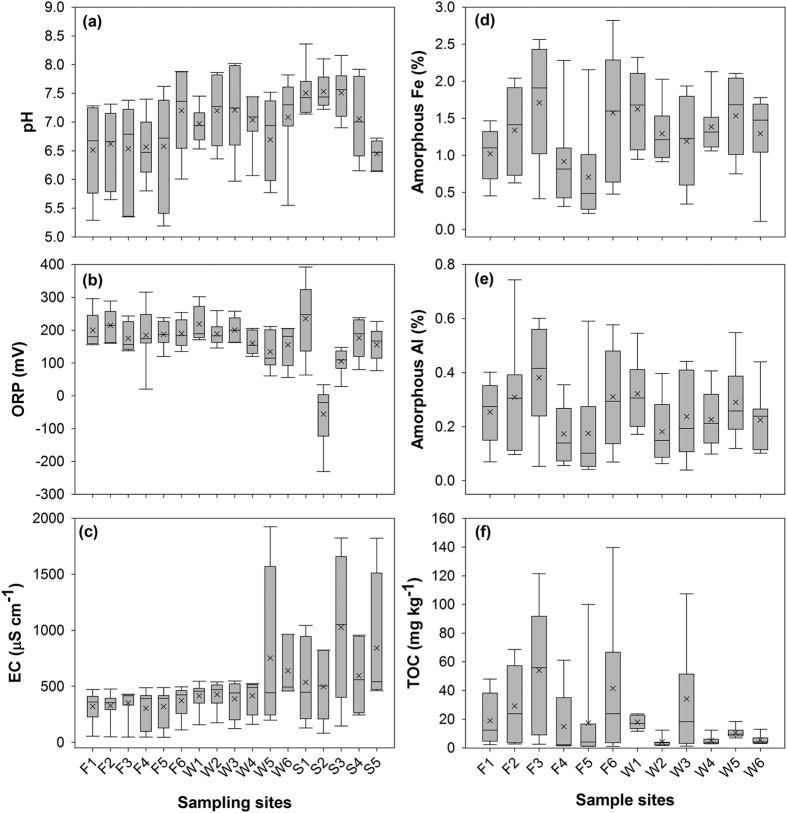
Environmental factors of water. (a) pH, (b) ORP, and sediments (c) EC, (d) amorphous Fe, (e) amorphous Al, and (f) TOC for the sampling sites. The pH, ORP, and EC data were collected from water samples. Amount of amorphous Fe, Al, and TOC were derived from sediment samples.

Our study sites locate at the basin of the Fazih and the Wu River in Taichung, Taiwan (Fig. 1). This 26-km basin has contained wastewater discharged from hospitals, the Taichung Industrial Park (traditional industrial activities), and the Central Taiwan Science Park (semiconductor industrial activities). Therefore, possible pollutants in this basin consist of emerging contaminants, heavy metals, and trace elements. The mean pH of individual sampling sites ranged from 6.5 to 7.5 (Fig. 2a). There was no significant variation in pH among all samples. Similar trend was found in the ORP (Fig. 2b). With the exception of the S2 sample, whose mean ORP is −55 mV, the mean ORP for other samples ranged from 134 to 235 mV. Coupled with the neutral pH level, the redox potential for all samples but S2 lay in the sub-oxic status, implying the redox potential was low enough to deplete O_2_ but not reduced SO_4_^13^. On the contrary, S2 was in an anoxic condition, wherein the redox potential was low enough to deplete SO_4_. For the EC, the sewage effluent samples (S1–S5) generally showed the relatively higher EC than that of the Fazih River (F1–F6) and most of the Wu River (W1–W4) samples (Fig. 2c). The mean EC for the sewage effluent samples ranged from 497 to 842 μS cm^−1^, resulting in a significant increase in EC for water samples collected at lower reaches of such effluents (W5 and W6). The enhancement in EC for the W6 sample might be also caused by the seawater intrusion as the sampling site of W6 locates close to the river mough.

Regarding chemical parameters of solid phases, we determined the contents of TOC and amorphous Fe and Al in sediments. For the amorphous (oxalate-extractable) Fe (0.71 to 1.71%) and Al (0.17 to 0.38%), no particular pattern could be identified according to the sampling geographical locations (Fig. 2d,e). However, TOC contents in sediments seemed to be divided into two groups associated with individual river basins (Fig. 2f). While the mean TOC contents for most sediment samples ranged from 15 to 54 mg kg^−1^, that for W2, W4, W5, and W6 sediments were less than 10 mg kg^−1^.

### Heavy metals and trace elements in river waters and sewage effluents

**Figure 3:**
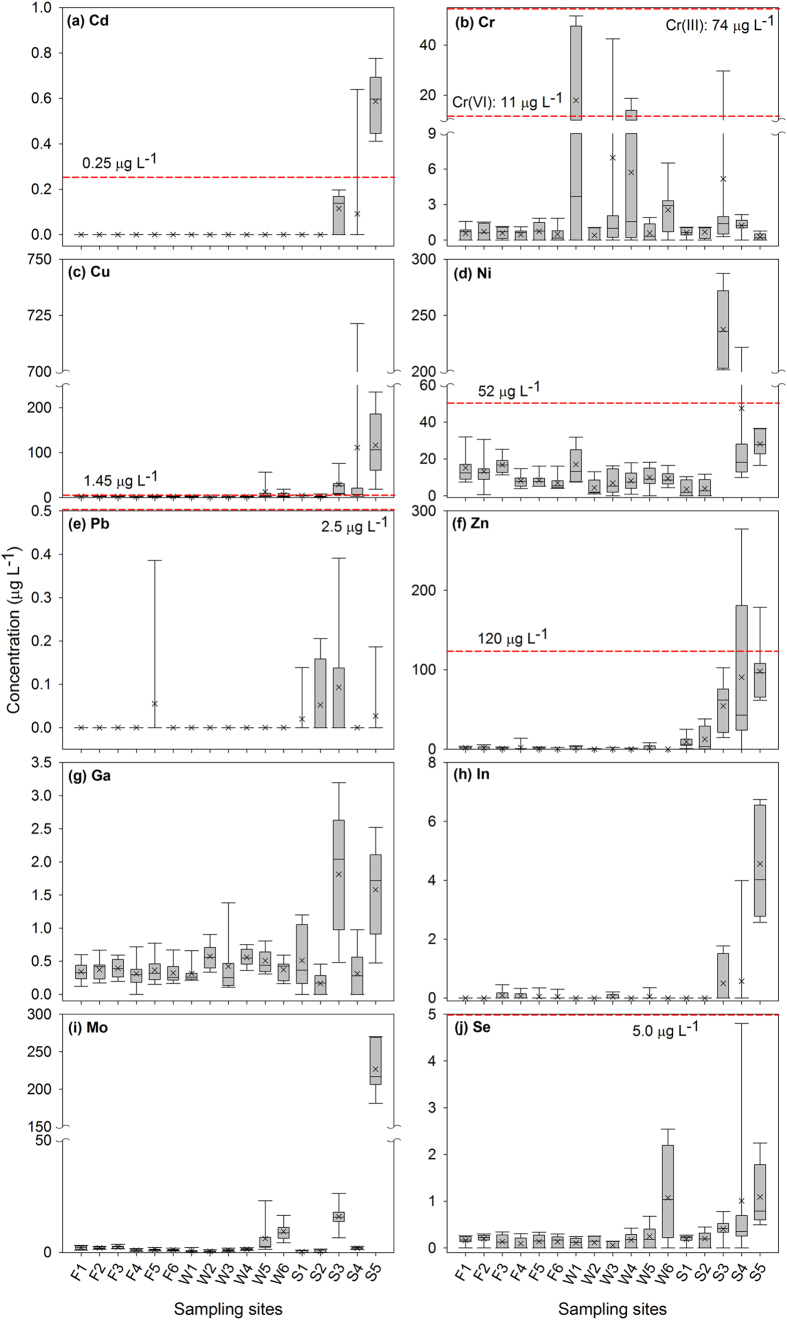
Metals contents for river waters sampled from the Fazih (F1~F6) and the Wu (W1~W6) River, and the sewage sampled from effluent outlets (S1–S5). Dash lines are values of the Criterion Continuous Concentration (CCC) established by the USEPA. Currently there is no CCC available for Ga, In, and Mo.

Figure 3 showed the maximum, mean, median, and minimum concentrations for Cd, Cr, Cu, Ni, Pb, Zn, Ga, In, Mo, and Se in river waters and sewage effluents. Metal concentrations were compared with the criterion continuous concentrations (CCC – dash lines in Fig. 3) established by the U.S. Environmental Protection Agency^14^. The CCC is an estimate of the highest concentration of a material in the surface water to which an aquatic community can be exposed indefinitely without resulting in an unacceptable effect^14^. Individual water samples generally displayed a wide difference in metal concentrations during the sampling events, indicating not only the temporal but the spatial variation among each sampling sites. With the exception of Cr, contents of heavy metals and trace elements in sewage effluents seemed relatively higher than that in the Fazih and the Wu River. While the highest mean concentration of Cd (0.59 μg L^−1^), Cu (116.35 μg L^−1^), Ni (237.60 μg L^−1^), Pb (0.09 μg L^−1^), Zn (98.01 μg L^−1^), Ga (1.81 μg L^−1^), In (4.56 μg L^−1^), Mo (226.64 μg L^−1^), and Se (1.09 μg L^−1^) was found in the site of S3 or S5, the highest mean amount of Cr (17.89 μg L^−1^) was found in the W1 site. Given that S3 and S5 were sewage effluents discharged mainly from the traditional and semiconductor industries, it is not surprising to observe substantial amounts of heavy metals in these samples. However, the occurrence of Cr in the W1, which exceeded the CCC, implying a peculiar source to Cr discharges.

### Heavy metals and trace elements in sediments

**Figure 4:**
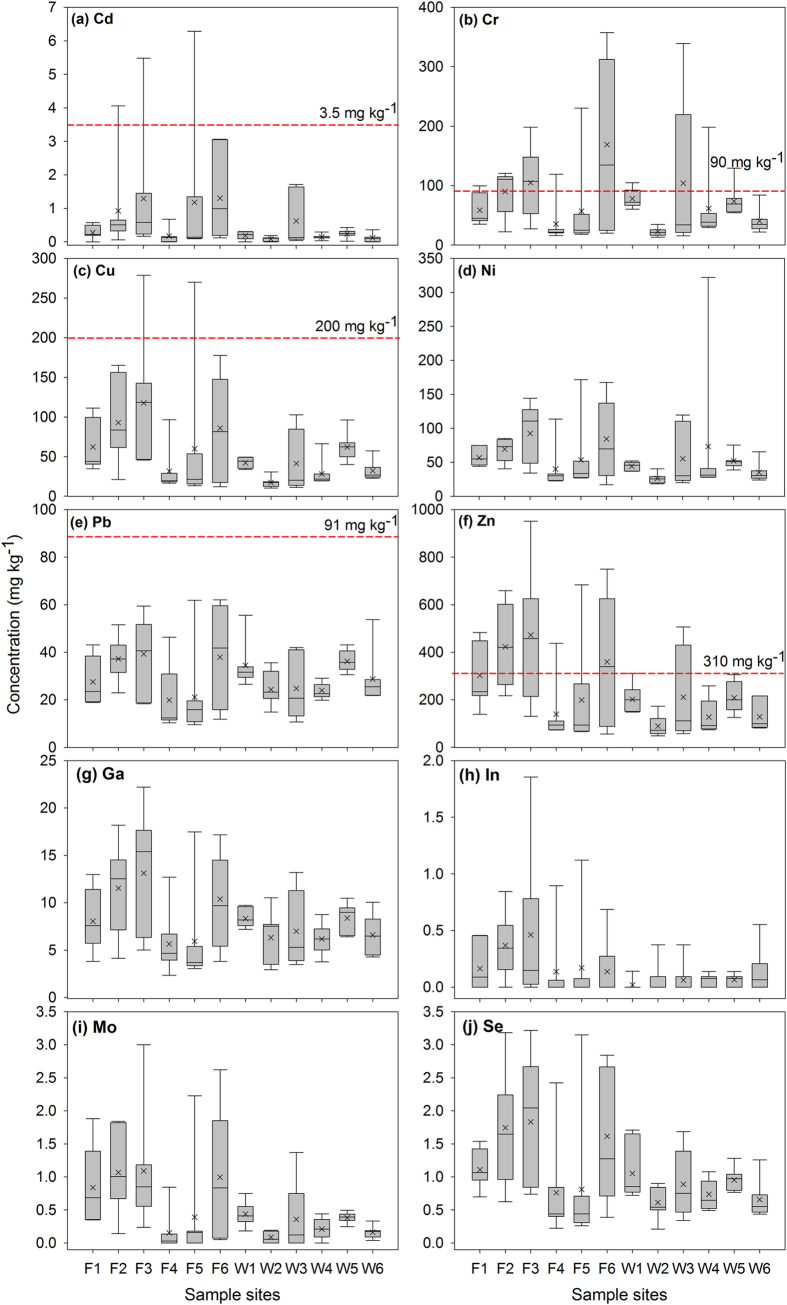
Metals contents of sediments sampled from the Fazih (F1~F6) and the Wu (W1~W6) River. Dash lines are values of the probable effect level (PEL) established by the Canadian Council of Ministers of Environment (CCME). Currently there is no PEL available for Ni, Ga, In, Mo, and Se.

The maximum, mean, median, and minimum concentration of heavy metals (Cd, Cr, Cu, Ni, Pb, Zn) and trace elements (Ga, In, Mo, Se) in sediments were summarized in Fig. 4. Metal concentrations were evaluated by the comparison with the probable effect level (PEL – dash lines in Fig. 4) established by the Canadian Council of Ministers of the Environment (CCME-15). Once metal concentrations exceed the PEL, adverse biological effects are expected to occur frequently. Such definition is based on the premise that the probability of toxic effects resulted from exposures to a given chemical increases with the concentration of that substance in sediments^15^,^16^.

Generally, sediments in the Fazih River seemed contain relatively higher amounts of heavy metals and trace elements than that in the Wu River. Plausible explanations include: (1) the Fazih River has contained the discharged sewage effluent from hospitals (S1 and S1) and traditional industrial activities (S3 and S4 – Fig. 1); (2) average volume rates of the water flow in the Fazih River (10.58 m^3^/s) is less than that in the Wu River (114.43 m^3^/s), resulting in a relatively limited mobilization of suspended particles and thereby a net deposition and accumulation of particulate metals in the Fazih River^17^; (3) the memory effect for metal accumulation from the previous sewage effluents discharged from the CTSP. The effluent outlet of the CTSP was relocated to the S5 site in December, 2009.

Although distribution of heavy metals and trace elements varied essentially among individual sites along the Fazih and the Wu River, the contamination of Cr and Zn is generally critical while compared with the PEL. For Cr, the highest level of 357.35 mg kg^−1^ was observed at the F6 site with the mean of 169.03 mg kg^−1^. In addition, sampling sites wherein both mean and median concentrations of Cr exceeded the PEL all located in the Fazih River (F2, F3, and F6). Similar trend was found for the Zn distribution. Only F2, F3, and F6 sites were exceeded in mean and median concentrations of Zn.

Given that the PEL for Ni, Ga, In, Mo, and Se has not been established yet^15^, we are not able to assess the environmental risk for such elements currently. However, among all tested trace elements, Se is particularly of interest to environmental scientists due to the narrow range between nutritionally required and toxic effect in organisms^18^. Literatures have strongly indicated that chronic Se toxicity could be triggered by the movement from the accumulation in the sediment into the food chain^19^. The threshold for biological toxic effects of Se ranged from 2 to 4 mg kg^−1^ 20,21, wherein the classification system of 20 that claimed the threshold of 2 mg kg^−1^ is more robust due to the more comprehensive computation for Se aquatic hazard assessments^22^. Such threshold was exceeded by the mean Se content of 2.05 mg kg^−1^ in the F3 sample.

### Ecological risk indices

Given that our study basin received pollution from multiple heavy metals, we used the quantitative approach developed by Hakanson^23^ to evaluate the potential ecological risk of heavy metal pollutions in sediments. The potential ecological risk index (*RI*) is defined as Equation (1).


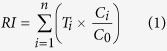


where *T*_i_ is the toxic-response factor for a given substance (e.g., Cd = 30, Cr = 2, Cu = Ni = Pb = 5, Zn = 1)^23^,^24^; *C*_i_ is the metal concentration in sediments; *C*_0_ is the regional background concentration of heavy metals, wherein we used the metal concentration obtained from the W2 site as the background because W2 received relatively less industrial and domestic sewage.

**Figure 5:**
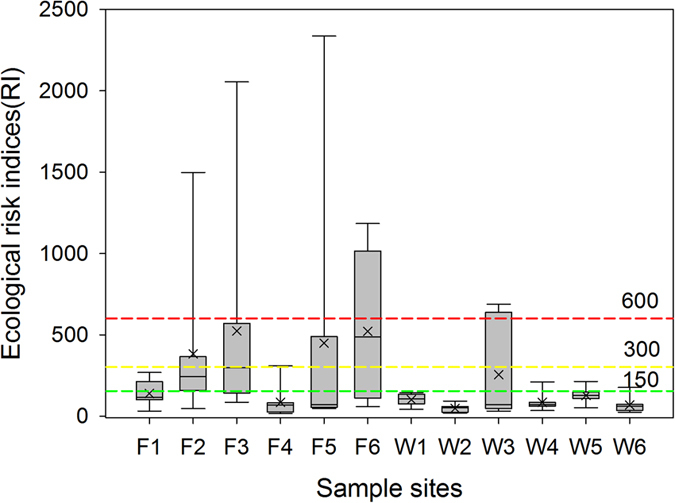
Ecological risk indices (*RI*) for sediments sampled from the Fazih (F1~F6) and the Wu (W1~W6) River. The *RI* values of 150, 300, and 600 are the boundaries to determine the potential ecological risk.

The *RI* of heavy metals in sediment samples were categorized into three zones as shown in Fig. 5. The following terminology was used to access the potential ecological risk for the basin indicated by the *RI* value^23^:


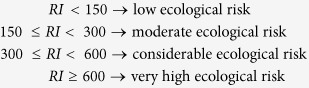


In our studied basin, the *RI* values for the Wu River sediments were generally lower than 300. However, four out of six sediment samples in the Fazih River showed the *RI* values ranging from 300 to 600, suggesting a considerable ecological risk in this basin. Given that the toxic response factor for trace elements such as Ga, In, Mo, and Se have not been developed well yet, the potential ecological risk of such trace elements were excluded here.

### Principal component analysis

**Figure 6:**
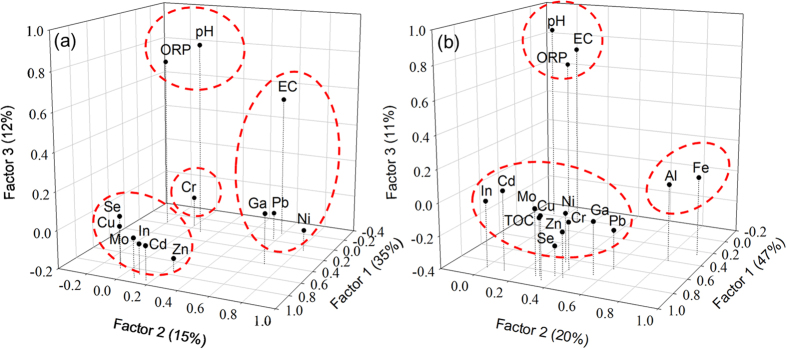
Plots of the PCA based on metal concentrations and environmental factors in (a) water and (b) sediment samples.

The PCA has been applied to determine the correlation between the partition of discharged metals and environmental factors such as pH, EC, ORP, TOC, and amorphous Fe and Al^7^,^8^,^25^,^26^,^27^. Results of PCA for metal contents in water samples and fluvial environmental factors of pH, EC, and ORP were showed in Fig. 6a. The PCA results suggested that heavy metals, trace elements, and environmental factors could be grouped into a four-component model, which accounted for 71.0% of the total variance (Tables S1 and S2 in Supplementary Materials). The first principal component that showed a contribution rate of 35.3% correlated with Cd, In, Mo, Cu, Se, and Zn. For the 2^nd^ component that accounted for 15.4% of variance, metals of Pb, Ni, and Ga were grouped into the environmental factor of EC. However, the other environmental factors of pH and ORP did not correlate with any metals (the 3^rd^ component with 12.0% of variance). Most noteworthily, Cr was isolated from all other metals and environmental factors (the 4^th^ component with 8.2% of variance). Such results implied that discharged heavy metals and trace elements could be categorized into three groups: (1) Cd, In, Mo, Cu, Se, Zn; (2) Pb, Ni, Ga; (3) Cr. In addition, the environmental factors of pH and ORP showed no correlation with the metal effluent. Only EC might influence the distribution of Pb, Ni, and Ga in river water.

For sediment samples, we added the contents of three more environmental factors including TOC, oxalate-extractable Fe and Al of individual sediment samples to process the PCA. According to Table S3 and S4 in Supplementary Materials, the matrix of 16 features (10 metals and 6 environmental factors) was grouped into a three-component model, describing approximately 77.7% of the total variance (Fig. 6b). The first component that described 46.9% of the total variance was highly loaded by all heavy metals, trace elements, and TOC. The amorphous Fe and Al were grouped together and represented as the second component with 19.6% of the total variance. For other environmental factors of pH ORP, and EC, they were classified as the third component, describing 11.2% of the total variance. In contrast to water samples, it is not able to classify metals in sediment samples into groups. All heavy metals and trace elements were correlated to TOC contents in sediments, implying OM mainly controlled the retention and further the distribution of metals in sediments. Neither solid-phase-related factors of amorphous Fe and Al nor solution-related factors of pH ORP, and EC showed the essential relationship for the metal retention in sediments.

### Retention of heavy metals and trace elements in sediments

By coupling the PCA results of water and sediment samples, the noteworthy finding for the metal distribution in our study river basin was that the correlation between discharged metals suggested by the PCA of water samples vanished in sediment samples. The disagreement in metal correlation between water and sediment samples is of interest to us in terms of (1) the rationale to use the metal contents in sediments for the identification of pollution sources^6^,^7^,^25^, (2) why the metal retention in sediments is highly correlated to TOC contents rather than other environmental factors, and (3) whether the metal species changes during the partition between solution-solid interfaces.

Widespread evidences have existed for the incorporation and retention of metals into OM and Fe-(hydr)oxides in soils and bed sediments^5^,^25^,^28^,^29^,^30^,^31^. In our study, however, the metal distribution in sediments only significantly correlated to the OM (TOC) contents. The OM has been widely recognized as a variable-charged material that consists of various functional groups such as carboxyl (R-COOH), phenolic OH (aromatic-OH), alcoholic OH (R-CH_2_-OH), and carbonyl (R-C = O-R-R/H/OR)^32^. Moreover, the OM also contains amine and amide groups as basic functional groups. Given that the dissociation constant (pK_a_) for carboxyl and phenolic groups ranges from 3–5 and 5–7, surfaces of natural OM are generally covered with negative charges while pH > 3^33^. In the contrary, surfaces of Fe-(hydr)oxides are often positively charged at neutral pH as their point of zero charge is generally >pH 8^34^,^35^,^36^. The species of metals would vary depending on the coupling of pH and redox potential in environments. With the exception of Se and Mo that occurred mainly as elemental species (Se_(s)_) or oxyanions (HSeO_3_^−^ and MnO_4_^2−^) under the environmental conditions of our study sites (Fig. 2), other metals generally presented as cations (Cu^+^/Cu^2+^, Ni^2+^, Zn^2+^, etc.) or hydrolyzed species (Cr(OH)^2+^, Pb(OH)^+^, etc.)^37^. Compared with Fe-(hydr)oxdies, thereby, most of metals might prefer to be retained by OM through the electrostatic attraction under circumambient environments. Similar trends in soils and sediments were reported by several scientists. Although Fe can stabilize metals by adsorption or coprecipitation with Fe-(hydr)oxides^29^,^30^,^31^, OM could act as a major sink for metals due to its strong complexing capacity^38^,^39^,^40^. For example, Grybos *et al*.^28^ evidenced that soil OM could be a more important sink for trace metals such as rare earth elements, Pb, and Ni than Fe-(hydr)oxides. The partition of Ni onto OM would increase with increasing pH^41^. For Cr and Cu, OM is also an important source although which cannot be quantified precisely due to the precipitation or re-adsorption^28^. In addition, Pan *et al*.^42^ also indicated that OM is the most important adsorption surface for the Cu, Cd, Pb, and Ni in paddy soils^42^.

Prior to the retention onto sediments, metals discharged from sewage effluents might transport in the form of soluble species in solutions and/or insoluble species in suspended solids. Taken into account that the metal concentrations in suspended particles were generally higher than that in bed sediments and/or even exceeded the PEL guidelines for the protection of aquatic life^43^, we were wondering whether any species transformations of metals in suspended solids occurred as the consequence of retention onto sediments. Here, Se speciation in suspended solids and sediments was performed as an example to examine the species transformation in relation to changes in environmental conditions.

### Selenium speciation

**Figure 7:**
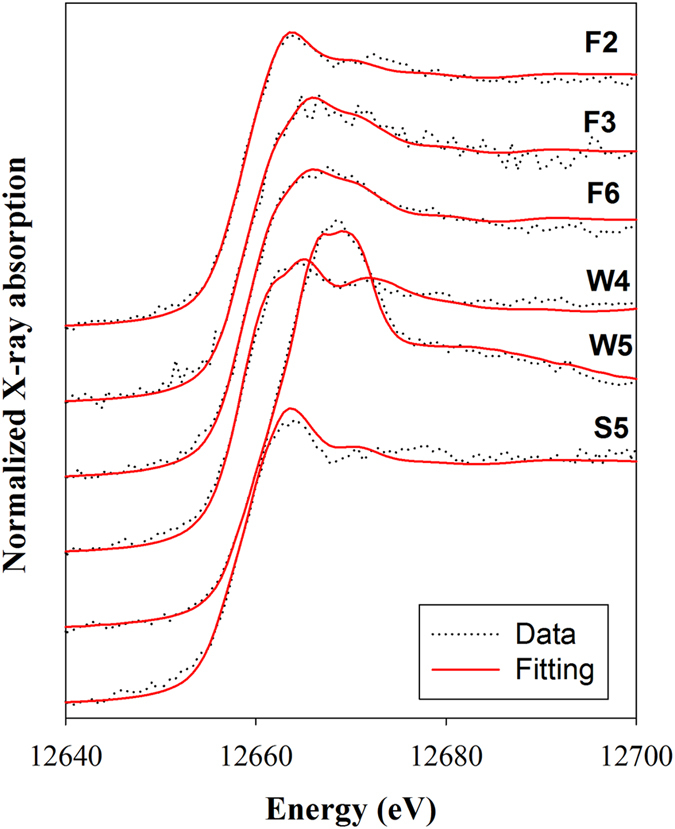
Selenium K-edge XANES spectra (dashed lines) and results of linear combination fitting (solid lines) for sediments collected in the Fazih (F2, F3, and F6) and the Wu (W4 and W5) River as well as suspension solids collected from the effluent outlet of S5.

Selenium speciation in selected suspended solids and sediments was performed using XANES analysis. Tested suspended solids and sediments were collected in January, 2015. Selenium concentrations for the Fazih River sediments (F2, F3, and F6) ranged from 2.17 to 3.18 mg kg^−1^, and that for the Wu River sediments (W4 and W5) were lower than 1 mg kg^−1^. However, in suspended solids (S5), the Se content was up to 65.2 mg kg^−1^, which was 20 to 60 times higher than that of sediments in the river basin. XANES data in Fig. 7 showed an essential spectral variation across all samples. According to the spectral features, the samples could be classified into three groups. The first one is the F2, W4, and S5, whose position of the white line peak located around 12663.5–12664.5 eV. The second group consists of F3 and F6 samples as they showed a similar white line peak at approximate 12666.7 eV. For the W5 samples, its spectral features significantly differed from other samples, wherein the position of the white line peak shifted to relatively higher energy at 12668.7 eV.

The presence and proportion of Se species in suspended solids and sediments were determined using LCF with spectra obtained from reference materials including iron selenide, elemental Se^0^, selenomethionine, selenocystine, trimethylselenonium, selenite, and selenate. Results of LCF shown as solid lines in Fig. 7 and Table 1 indicated that the Se inventory in all samples was dominated mainly by FeSe (>57%). Such selenide could be originated from materials like CdSe and ZnSeused in semiconductor industries^44^. For the F2 and S5 samples, the elementary Se^0^ contributed about 38% of the total Se besides the FeSe. However, in the samples of F3, F6, and W4, not only inorganic FeSe but organic Se of trimethylselenonium was found. The most concerned point is that Se oxyanions (selenite and selenate) was observed at the W5 site, wherein the sediment received a massive amount of Se from the suspended solids of S5. Selenite and selenate are highly soluble, hence more mobile than the less soluble forms such as selenide and elemental Se^0^ 45. In addition, selenite poses an approximately 10-fold higher bioaccumulation factor than selenate for the phytoplankton Se uptake^46^. In term of toxicity, the relative sequence for individual Se species is: selenite >selenate >selenide >elemental Se^0^^47^. Although organo-Se are less soluble, they are relatively readily absorbed by organisms^48^. To sum up, the apparent discrepancy in Se species over the course of transportation processes may be caused by the various environmental conditions between waters and sediments. Hence, a further study to determine the transformation of metal species in relation to various environmental conditions is merited for undertaking environmental risk assessments and understanding metal distribution and transfer into the food chain in ecosystems.

## Discussion

One of the major environmental impacts for the metal accumulation in sediments was the metal uptake by primary producers and the subsequent access into the food webs of aquatic ecosystems. In this study, the distribution and accumulation of heavy metals and trace elements in relational to environmental factors in the basin of the Fazih and the Wu River that received sewage effluents from traditional and semiconductor industrial activities was determined by PCA. The PCA results pointed out the discrepancy in metal distributions between water and sediment samples. Although PCA for water samples proposed three potential origins of contamination for heavy metals and trace elements, such metal distribution was not observed while metals accumulated in sediments. Among all tested environmental factors, the metal distribution in sediments only correlated to OM contents. In spite of other environmental factors such as pH and ORP did not directly influenced the metal distribution in sediments, such physical-chemical properties in environments may control the species transformation for metals and further change their mobility, bioavailability, and toxicity. Our XANES results indicated that Se inventory mainly comprised selenide and elemental Se^0^ in suspended solids collected from sewage effluents. However, selenite and selenate was found in the sediment received a massive amount of Se from suspended solids. This is of environmental concern as selenite and selenate could bioaccumulate in organisms and thus incorporate into food webs more readily than selenide and elemental Se^0^.

Collectively, our results clearly suggest that OM must be taken into account to assess the metal behavior in sediments. Recognition of the roles of environmental factors in metal distribution could lead to a better quantification in dynamics and mass balances for metals in contaminated ecosystems. Given that bed sediments serve both as a sink and source of metals, the prediction of relative abundance of metals in sediments could improve strategies for the management and remediation of metal contamination. Conclusively, the consideration of environmental factors, especially OM, is required while monitoring metal accumulation in sediments. Such comprehensive information can help to develop an adequate protection and restoration plan.

## Methods

### Study areas and sample collection

The water and sediment samples were collected from 12 sampling sites scattered in the Fazih (F1–F6) and the Wu (W1–W6) River (Fig. 1). Sewage effluents discharged from hospitals (S1 and S2), the Taichung Industrial Park (S3), the Taichung Precision Machinery Innovation Technology Park (S4), and the Central Taiwan Science Park (CTSP, S5) were also collected. There were seven sampling events during June, 2014 to August, 2015.

Water samples were collected at river surfaces or from sewage effluents. The pH, EC, and ORP data of water samples were determined using a multi-parameter water quality checker (Horiba U-50) immediately after the collection. Once in the laboratory, the other portion of water samples was filtered through a 0.2 μm membrane, acidified to a 2% HNO_3_ background, and preserved in acid-washed polyethylene bottles at 4 °C. For sediments, approximately 100 g of solids were collected using a plastic scoop from the top 2 cm of bottom sediments and preserved in acid-washed polyethylene containers at 4 °C. Sediment samples were air-dried inside a laminar flow chamber, passed through a 2-mm nylon sieve, and ground in an agate mortar. For elemental analysis, 1 g of pretreated sediment was acid digested using 10 mL of aqua regia at 180 °C for 4 h. Metal concentrations in acid digestates were determined by inductively coupled plasma mass spectrometry (ICP-MS, Elan DRC II, PerkinElmer) with methane as reaction gas to reduce interatomic interferences. A soil standard reference material (SRM, CRM029 from Sigma-Aldrich) was also digested to check the analysis recovery. Three replicates were performed in acid digestion for each sample including blank and SRM for quality control. The recoveries of the SRM ranged from 80 to 120% in all metals. Contents of OM in sediments were determined by amounts total organic carbon (TOC), which was determined using the TOC analyzer (Multi N/C 2100, Analytik Jena). Sediment samples for TOC analysis were pretreated using 10% phosphoric acid overnight to reduce the interference from inorganic carbon^49^,^50^. The amorphous Fe and Al contents in sediments were determined using acid ammonium oxalate extraction method^51^. Briefly, 20 mL of 0.2 M acidified ammonium oxalate was added to 0.5 g of pretreated sediments. Suspensions were shaken horizontally in darkness for 2 h and then centrifuged. The Fe and Al amounts in supernatant were determined using the ICP-MS.

### Principal component analysis

Principal component analysis (PCA) has been often used to interpret the multivariate, complex, redundant, and not direct data^52^,^53^,^54^. Here, we used PCA to determine the correlation of metal discharges and to understand the role of environmental factors that affect the metal retention in sediments. The PCA reduces variables into a set of principal components and gives a new multidimensional system. The PCA and correlation analysis were performed using the SPSS 19.0 software (IBM^®^) for the Windows. To interpret the results more precisely, the rotation method - Varimax with Kaiser Normalization - was applied to maximize variances of the squared normalized factor loadings across variables for each factor. All principal factors extracted from variables were those whose eigenvalues is higher than 1^55^.

### Data collection and analysis for Se K-edge XANES spectroscopy

Selenium speciation in selected sediments and suspended solids was determined using Se K-edge XANES analysis. Suspended solids were collected by filtering the sewage effluent at the S5 site thought a mixed cellulose esters membrane with a calibrated porosity of 0.2 μm (Millipore). The filter membrane was placed on a standing stainless steel filter holder (90 mm, Millipore) and the filtration was operated under an air pressure of 65 psi. All samples for Se-XANES analysis were prepared by mounting approximately 0.2 g solids in acrylic sample holders, sealing with Kapton tape to avoid desiccation. Spectra were acquired at the Beamline BL17C1 at the National Synchrotron Radiation Research Center (NSRRC), Hsinchu, Taiwan. The linear combination fitting (LCF) of Se-XANES data across from 30 eV below to 40 eV above the Se absorption edge was used to determine the Se species. Reference materials used in LCF included iron selenide (FeSe), elemental (gray) Se^0^, selenomethionine, selenocystine, trimethylselenonium, selenite (H_2_SeO_3_), and selenate (H_2_SeO_4_). See Liu, *et al*.^45^ for additional details in data collection and processing.

## Additional Information

**How to cite this article**: Hsu, L.-C. *et al*. Accumulation of heavy metals and trace elements in fluvial sediments received effluents from traditional and semiconductor industries. *Sci. Rep.* **6**, 34250; doi: 10.1038/srep34250 (2016).

## Supplementary Material

Supplementary Information

## Figures and Tables

**Table 1:** Linear combination fitting results for Se K-edge XANES spectra of sediments collected in the Fazih (F2, F3, and F6) and the Wu (W4 and W5) River as well as suspension solids collected from the effluent outlet of S5.

Sample name	Se (mg Kg^−1^)	FeSe (mol%)^b^	Elemental Se^0^ (mol%)^b^	Trimethyl- selenonium (mol%)^b^	Selenite (mol%)^b^	Selenate (mol%)^b^	*R-factor*^c^ (×10^3^)
F2	3.18 ± 0.12	60.7 ± 2.3	39.3 ± 2.3				1.64
F3	2.17 ± 1.64	76.8 ± 3.3		23.2 ± 3.3			4.78
F6	2.84 ± 0.38	79.8 ± 1.9		20.2 ± 1.9			1.40
W4	0.94 ± 0.08	57.3 ± 1.8		42.7 ± 1.7			2.44
W5	0.97 ± 0.06	58.2 ± 1.3			27 ± 0.9	14.8 ± 0.7	1.37
S5	65.2 ± 4.11	61.8 ± 3.3	38.2 ± 3.3				2.96

The data show the proportion (in units of mol%) of the reference spectra that resulted in the best fit to the sample data^a^.

^a^Samples tested for Se-XANES analysis were collected in January, 2015.

^b^Mean ± standard deviation. The weighting factors on each fit were summed to 100 ± 1% and were normalized to 100%.

^c^Normalized sum of the squared residuals of the fit (*R-factor* =  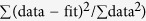.
