# Supplementary material for: Accumulation of heavy metals and trace elements in fluvial sediments received effluents from traditional and semiconductor industries
Source: Sci Rep. 2016 Sep 29;6:34250. doi: 10.1038/srep34250 (PMC5041097; doi:10.1038/srep34250)
Supplement: Supplementary Information [file srep34250-s1.pdf]

Supplementary Materials

Accumulation of heavy metals and trace elements in fluvial sediments received effluents from traditional and semiconductor industries

Liang-Ching Hsu[a], Ching-Yi Huang[b], Yen-Hsun Chuang[b], Ho-Wen Chen[b], Ya-Ting Chan[c], Heng Yi Teah[d], Tsan-Yao Chen[e], Chiung-Fen Chang[b], Yu-Ting Liu[c]*, Yu-Min Tzou[c]*

[a] Scientific Research Division, National Synchrotron Radiation Research Center, 101 Hsin-Ann Road, Hsinchu 300, Taiwan (R.O.C.)

[b] Department of Environmental Science and Engineering, Tunghai University, 1727 Sec.4, Taiwan Boulevard, Taichung 407, Taiwan (R.O.C.)

[c] Department of Soil and Environmental Sciences, National Chung-Hsing University, 145 Xingda Rd., Taichung 402, Taiwan (R.O.C.)

[d] Division of Environmental Studies, Graduate School of Frontier Sciences, The University of Tokyo, 332 Building of Environmental Studies, 5-1-5 Kashiwanoha, Kashiwa City, Chiba 277-8563, Japan

[e] Department of Engineering and System Sciences, National Tsing Hua University, No. 101, Section 2, Kuang-Fu Road, Hsinchu 300, Taiwan, R.O.C.

*Corresponding author:

Yu-Ting Liu: email: yliu@nchu.edu.tw; TEL: +886-4-2284-0373 ext. 3402; Fax: +886-4-2285-6050

Yu-Min Tzou: ymtzou@dragon.nchu.edu; Tel.: +886-4-2284-0373 ext. 4206; fax: +886-4-22855167

Table S1. Total variance explained for the heavy metals and trace element in water samples.

Component	-------- Initial eigenvalues --------			-- Extraction sums of squared loadings --			-- Rotation sums of squared loadings --		
	Total	Variance (%)	Cumulative (%)	Total	Variance (%)	Cumulative (%)	Total	Variance (%)	Cumulative (%)
1	4.74	36.46	36.46	4.74	36.46	36.46	4.59	35.32	35.32
2	1.95	15.02	51.47	1.95	15.02	51.47	2.01	15.43	50.75
3	1.50	11.57	63.05	1.50	11.57	63.05	1.57	12.04	62.79
4	1.04	7.97	71.02	1.04	7.97	71.02	1.07	8.23	71.02
5	0.93	7.14	78.16						
6	0.77	5.93	84.08						
7	0.72	5.55	89.63						
8	0.48	3.68	93.31						
9	0.37	2.84	96.15						
10	0.23	1.74	97.89						
11	0.16	1.26	99.15						
12	0.09	0.65	99.80						
13	0.03	0.20	100.00						

[a] Extraction method: principal component analysis.

Table S2. Component matrices explained for heavy metals and trace elements in water samples.

	-------- Rotated component matrix --------			
Elements	F1	F2	F3	F4
Cd	0.941	0.166	-0.022	-0.022
Pb	-0.032	0.577	-0.055	-0.218
In	0.876	0.087	-0.041	-0.004
Mo	0.931	0.081	0.004	-0.059
Ni	0.090	0.827	-0.089	0.212
Cu	0.838	-0.065	0.021	-0.015
Ga	0.410	0.693	0.068	0.070
Se	0.785	-0.094	0.056	0.010
Zn	0.742	0.245	-0.134	0.018
Cr	-0.049	-0.004	-0.038	0.916
pH	0.043	0.085	0.838	-0.221
ORP	-0.033	-0.200	0.720	0.243
EC	-0.146	0.588	0.551	-0.142

[a] Extraction method: principal component analysis.
[b] Rotation method: varimax with Kaiser normalization.
[c] Rotation converged in 5 iterations.

Table S3. Total variance explained for heavy metals and trace elements in sediment samples.

Component	-------- Initial eigenvalues --------			-- Extraction sums of squared loadings --			-- Rotation sums of squared loadings --		
	Total	Variance (%)	Cumulative (%)	Total	Variance (%)	Cumulative (%)	Total	Variance (%)	Cumulative (%)
1	9.50	59.35	59.35	9.50	59.35	59.35	7.51	46.93	46.93
2	1.76	11.00	70.35	1.76	11.00	70.35	3.13	19.55	66.48
3	1.18	7.36	77.71	1.18	7.36	77.71	1.80	11.23	77.71
4	0.83	5.18	82.89						
5	0.71	4.46	87.36						
6	0.53	3.33	90.69						
7	0.33	2.03	92.72						
8	0.31	1.90	94.63						
9	0.23	1.45	96.07						
10	0.17	1.08	97.15						
11	0.17	1.05	98.20						
12	0.11	0.70	98.90						
13	0.06	0.39	99.29						
14	0.05	0.33	99.61						
15	0.04	0.24	99.85						
16	0.02	0.15	100.00						

[a] Extraction method: principal component analysis.

Table S4. Component matrices explained for heavy metals and trace elements in sediment samples.

	----- Rotated component matrix ------		
Elements	F1	F2	F3
Cd	0.884	0.109	0.082
Pb	0.631	0.609	-0.162
In	0.884	0.008	0.005
Mo	0.881	0.289	0.003
Ni	0.646	0.342	-0.097
Cu	0.918	0.340	-0.018
Ga	0.746	0.548	-0.078
Se	0.838	0.377	-0.220
Zn	0.843	0.423	-0.127
Cr	0.737	0.405	-0.109
TOC	0.871	0.308	-0.051
Fe	0.256	0.928	0.085
Al	0.401	0.819	0.071
pH	-0.029	-0.075	0.850
ORP	-0.105	-0.015	0.615
EC	0.025	0.106	0.746
Cd	0.884	0.109	0.082

[a] Extraction method: principal component analysis.
[b] Rotation method: varimax with Kaiser normalization.
[c] Rotation converged in 4 iterations.
